# A computational fluid dynamics analysis on Fe_3_O_4_–H_2_O based nanofluid axisymmetric flow over a rotating disk with heat transfer enhancement

**DOI:** 10.1038/s41598-023-31734-1

**Published:** 2023-03-22

**Authors:** Umar Farooq, Ali Hassan, Nahid Fatima, Muhammad Imran, M. S. Alqurashi, Sobia Noreen, Ali Akgül, Abdul Bariq

**Affiliations:** 1 Department of Mathematics, Government College University Faisalabad, Faisalabad, 38000 Pakistan; 2 Department of Mathematics, University of Gujrat, Gujrat, 50700 Pakistan; 3 Department of Mathematics and Sciences, Prince Sultan University, Riyadh, 11586 Saudi Arabia; 4 Department of Mathematics and Statistics, College of Science, Taif University, P.O. Box 11099, Taif, 21944 Saudi Arabia; 5 Department of Chemistry, Government College Women University Faisalabad, Faisalabad, 38000 Pakistan; 6 Department of Computer Science and Mathematics, Lebanese American University, Beirut, Lebanon; 7 Art and Science Faculty, Department of Mathematics, Siirt University, 56100 Siirt, Turkey; 8 Mathematics Research Center, Department of Mathematics, Near East University, Near East Boulevard, 99138 Nicosia/Mersin 10, Turkey; 9 Department of Mathematics, Laghman University, Laghman, 2701 Afghanistan

**Keywords:** Engineering, Mathematics and computing, Physics

## Abstract

In present times modern electronic devices often come across thermal difficulties as an outcome of excessive heat production or reduction in surface area for heat exclusion. The current study is aimed to inspect the role of iron (III) oxide in heat transfer enhancement over the rotating disk in an axisymmetric flow. Water is utilized as base fluid conveying nano-particle over the revolving axisymmetric flow mechanism. Additionally, the computational fluid dynamics (CFD) approach is taken into consideration to design and compute the present problem. For our convenience, two-dimensional axisymmetric flow configurations are considered to illustrate the different flow profiles. For radial, axial, and tangential velocity profiles, the magnitude of the velocity, streamlines, and surface graphs are evaluated with the similarity solution in the computational fluid dynamics module. The solution of dimensionless equations and the outcomes of direct simulations in the CFD module show a comparable solution of the velocity profile. It is observed that with an increment in nanoparticle volumetric concentration the radial velocity decline where a tangential motion of flow enhances. Streamlines stretch around the circular surface with the passage of time. The high magnetization force $0 \le {{\mathrm{m}}_{1}}\le 6$ resist the free motion of the nanofluid around the rotating disk. Such research has never been done, to the best of the researchers’ knowledge. The outcomes of this numerical analysis could be used for the design, control, and optimization of numerous thermal engineering systems, as described above, due to the intricate physics of nanofluid under the influences of magnetic field and the inclusion of complex geometry. Ferrofluids are metallic nanoparticle colloidal solutions. These kinds of fluids do not exist in nature. Depending on their purpose, ferrofluids are produced using a variety of processes. One of the most essential characteristics of ferrofluids is that they operate in a zero-gravity environment. Ferrofluids have a wide range of uses in engineering and medicine. Ferrofluids have several uses, including heat control loudspeakers and frictionless sealing. In the sphere of medicine, however, ferrofluid is employed in the treatment of cancer via magneto hyperthermia.

## Introduction

Nano-particles are useful for a variety of diagnostic purposes as well as the successful treatment of cardiovascular disorders. Physical features of nano-particles, including shape, surface, and size alterations, provide improved treatment options, and these physical qualities are largely responsible for nano-particle distribution into the artery. Magnetic nanoparticles are suspended in colloidal solutions called Ferro-fluids. These fluids don't exist in the natural world. Depending on the purpose, Ferro-fluids are made in a variety of ways. Ferro-fluids function in a zero-gravity environment, which is one of its most essential features. Ferro-fluids offer a wide range of uses in engineering and medicine. Ferro-fluids are used in a wide range of applications, including heat control loudspeakers and seamless sealing. Ferro-fluid, on the other hand, is employed in the medical profession to cure cancer using magneto hyperthermia. Ferro-fluids are colloidal suspensions of ultrafine (3–10 nm) single-domain magnetic nano-particles that may modify their physical characteristics when a magnetic field is applied^1,2^. Their uses are many, spanning industries as diverse as defense, space, refrigeration, and medical. Light scattering, thermal conductivity tests, rheology, critical angle neutron diffraction, and Brownian dynamics have all been used to investigate field-induced modifications in the characteristics of ferrofluid.

Sheikholeslami et al.^3^ investigated the significance of nanofluid conveying alumina nano-particles in a porous medium using Darcy law. Bhandari et al.^4^ discussed the flow of water-based nanofluid using nano-particles between two rotating disks with variable viscosity and thermal conductivity. Shojaeizadeh et al.^5^ conducted an experimental study to investigate the magneto-viscous effect on magnetic nanofluid under magnetic field influence. Alsarrafet al.^6^ explored the effect of the magnetic field on convective heat transfer in the heated tube using hybrid nanofluids. Dogonchi and Ganjii^7^ explained the thermal radiation impact on heat transfer in nanofluid over a stretching surface with Brownian motion. Rashad et al.^8^ described magneto-hydrodynamic natural convection in a squared cavity analyzing the effect of source-sink size and location. Parvin et al.^9^ explained variable thermal conductivity impact on the natural convection of nanofluids in an annulus. Some noticeable works on nanofluids are given for knowledge purposes^10,11^. Experimental and theoretical study on Ferro-fluid has risen in recent years and has resulted in several engineering and medicinal implications. Ferro-fluids are nanoscale colloidal suspensions. A chemical technique can be used to create it. One of the most common uses of Ferro-fluid is to seal hard disc drives. It boosts the acoustic output of loudspeakers while preventing the coil from overheating. It may also be used as a lubricant as well as a coolant. Ferro-fluids, on the other hand, are crucial in cancer treatment using magnetic hyperthermia in the medical profession. In the absence of gravity, Ferro-fluid may flow. This section presents a literature overview of current developments in heat and mass transfer calculations on Ferro-fluid flow. Dynamic sealing, electronics industries, computer hardware, aircraft, loudspeakers, and bioengineering all employ Ferro-fluids^12,13^. Salawu et al.^14^ discussed the effect of magnetization force, variable viscosity, and thermal conductivity on the swirling flow Von Karman flow of ferromagnetic nanofluids over-stretching spinning disks. Hussain et al.^15^ studied compressible flow in the rectangular cavity using the computational fluid dynamics approach. Arshad and Hassan^16^ elaborated on hybrid nanofluids for heat transfer between permeable rotating disks. Cummins et al.^17^ elaborated flow past permeable disks at low Reynolds numbers. Bilal et al.^18^ examined the shape factor effect on hybrid nanofluid between permeable disks using a single-phase flow model. Bashir et al.^19^ analyzed thermophoresis phenomena between rotating disks under the thermal radiation effect. Animasaun et al.^20^ studied magnetic flux density and heat source/sink effects on ternary hybrid nanofluids dynamics over the convectively heated boundary. Heat transfer enhancement is an important problem in various industries such as solar collectors, heat exchangers, and MEMS. The addition of metallic or nonmetallic nanometer (nm) sized particle in host base fluid (water, oils, ethylene glycol) boost the thermal conductivity of these fluids. Consequently, heat transmission increases. Over the past decade instead of one two nano-particle are distributed into the base fluids to form hybrid nanofluids to increment the thermal conductivity of fluids. Bhandari et al.^21^ discussed the heat transfer enhancement using ferrofluid over a stretching surface under the static magnetic influence. Jalili et al.^22^ investigated heat and mass transfer of ferrofluid under suction and injection effect. Gowda et al.^23^ explored the application of Stefan suction on ferrofluid under magnetic dipole effect for heat transfer enhancement. Animasaunet al.^24^ in their new book provided comprehensive scrutinization and analysis of the ratio of momentum diffusivity to thermal diffusivity. Elnaqeeb et al.^25^ examined shape effect and suction influence on the three-dimensional flow of ternary hybrid nanofluids in the event of dual stretching. Saleem et al.^26^ elaborated thermo-migration and Brownian motion over the horizontal surface using three distinct particles distributed in base water fluid. Cao et al.^27^ discussed different levels of partial slip effect on ternary hybrid nanofluid utilizing different nanoparticles in base water fluid. Acharya^28^ investigated the effects of hybrid nanofluid flow with thermal boundary conditions over a circular cylinder. Acharya^29^ studied the effects of ferrofluid flow with nanoparticles diameter and solid–liquid via a spinning disk. Acharya^30^ analyzed the effects of nanofluid with nanoparticles with thermal radiation via a spinning disk. Acharya^31^ investigated the radiative nanofluid spraying over a permeable inclined spinning disk with a heat source/sink. Acharya^32^ investigated the effects of nanoparticle diameter and nanolayer on the ferrofluid flow over a slippery rotating disk. Acharya et al.^33^ analyzed the aspects of entropy generation optimization of unsteady radiative hybrid nanofluid flow over a slippery spinning disk. Acharya et al.^34^ studied the variations of nanofluid flow by the influence of nanoparticles' diameter. Acharya^35^ expressed the simulation of the flow patterns and thermal control of radiative nanofluid spraying on an inclined revolving disk. In the above literature review, the significance of mono, hybrid, and ternary nanofluids is illustrated in heat transfer enhancement and other such engineering applications. According to the works^20,25–27^, ternary hybrid nanofluid transmission is magnificently influenced by thermo-migration and Brownian motion and distinct convection phenomena.

In this work the significance of ${\mathrm{Fe}}_{2}{\mathrm{O}}_{3}$ nano-particles heat transfer enhancement in the flow over a rotating disk. The flow governing model equations is achieved using the in-compressible system of Naiver-Stokes in the presence of externally applied body force effect of magnetization. To demonstrate the outcomes of the study more effectively two-dimensional plot setting has been taken into account. The model has been designed and computed in the COMSOL-Multiphysics. Radiation and tangential components of velocity, pressure contours, streamlines pattern, change in magnitude of velocity through 3-dimensional plots, and change in velocity magnitude on the surface of the disk have been elaborated over time. Additionally, the line graph is presented to depict the out of change in motion of nanofluid around the rotating disk over the varying time. Furthermore, the finite element method has been utilized for simulations.

## Mathematical Formulation

**Figure 1:**
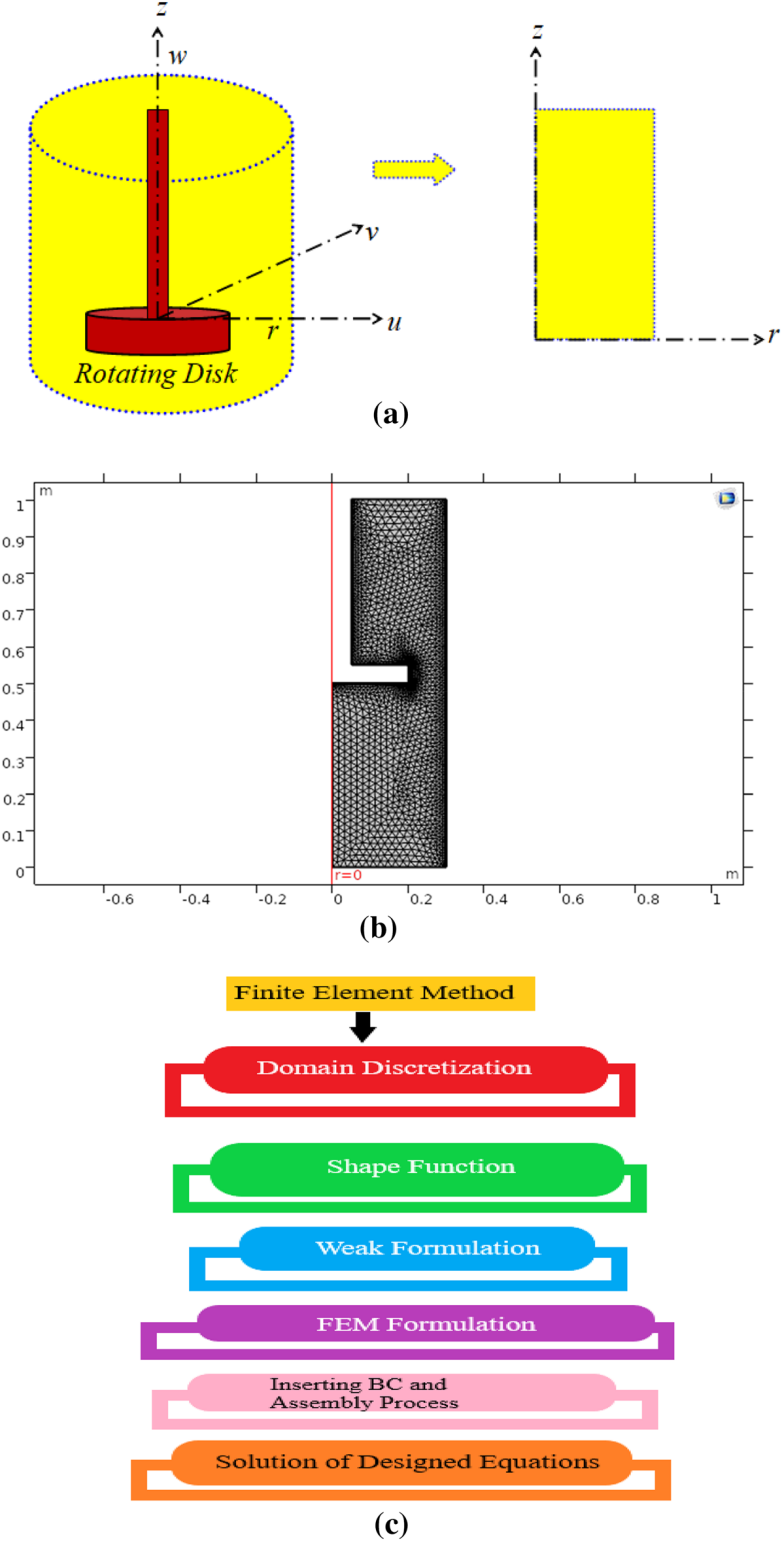
**(a)** Flow geometry of the problem, **(b)** rotating disk two-dimensional CFD domain mesh, **(c)** steps involved in the FEM procedure.

Figure 1a shows a schematic illustration of the flow caused by a revolving disk in a tank. The disc spins evenly about the z-axis, generating a 3D boundary layer. Because the flow is axisymmetric, the 3D flow has been evaluated across a two-dimensional area in the CFD module. The radial and axial velocities of the water-carrying $\left( {{\text{Fe}}_{3} {\text{O}}_{4} } \right)$ nanofluid vary. The nano-particle is selected to constitute our desired nanofluid due to its vast application in science and engineering. In a recent experimental study, Shah et al.^36^ discussed the significance of water-based nanofluid $\left( {{\text{Fe}}_{3} {\text{O}}_{4} } \right)$ for nano-coolant. Srinivasan et al.^37^ used engine oil-based nanofluid for heat transfer enhancement in the plate using $\left( {{\text{Fe}}_{3} {\text{O}}_{4} } \right)$ nano-particle. Afshari et al.^38^ studied heat transfer enhancement in finned shells and tubes using water-based nanofluid and they were employed $\left( {{\text{Fe}}_{3} {\text{O}}_{4} } \right)$ as a working nano-particle. The similarity technique is applied in Fig. 1a for 3D geometry. The computational domain in the CFD module is the two-dimensional geometry. These two rotating flow techniques for velocity profiles are contrasted.

The flow of magnetic fluid is described as ^4,13^:

1 $$ \nabla \cdot V = 0 $$

2 $$ \rho_{n} \left[ {V_{t} + \left( {V \cdot \nabla } \right)V} \right] = - \nabla p + \mu_{0} M\nabla H + \mu_{n} \nabla^{2} V + \frac{1}{{2\tau_{s} }}\nabla \times \left( {\omega_{p} - \Omega } \right) $$

3 $$ I\frac{{d\omega_{p} }}{dt} = \mu_{0} \left( {M \times H} \right) - \frac{1}{{\tau_{s} }}\left( {\omega_{p} - \Omega } \right) $$

Here the inertial term $\left( {I\frac{{d\omega_{p} }}{dt}} \right)$ is negligible to the relaxation term $\left( {I\frac{{\omega_{p} }}{{\tau_{s} }}} \right)$.

4 $$ \omega_{p} = \Omega + \mu_{0} \frac{{\tau_{s} }}{I}\left( {M \times H} \right) $$

By (4), Eq. (2) is written as

5 $$ \rho_{n} \left[ {V_{t} + \left( {V.\nabla } \right)V} \right] = - \nabla \tilde{p} + \mu_{n} \nabla^{2} V + \frac{{\mu_{0} }}{2}\nabla \times \left( {M \times H} \right) $$

The equilibrium of the magnetic and viscous torque can be written from (5) as:

6 $$ \mu_{0} \left( {M \times H} \right) = - 6\mu_{n} \varphi_{1} \left( {\Omega - \omega_{p} } \right) $$

Barci et al.^39^ has solved the difference relation on the right-hand side of Eq. (6). He suggested that a constant field always impedes free particle rotation. Thus, inverse inequalities are impossible in thermodynamics. They achieved the following relation using rotational velocity relation and angular velocity of particles ${\omega }_{p}=(1-g)\Omega$. The derivation is already done in ^39^ so we must omit it. The following relation has been obtained for magnetic torque^13,40^:

7 $$ \mu_{0} \left( {\overline{M \times H} } \right) = - 6\mu_{n} \varphi_{1} m_{1} \Omega $$

The effective magnetic number $\left( {m_{1} } \right)$ owing to the applied magnetic field. From Eqs. (6) and (7)

8 $$ \frac{{\mu_{0} }}{2}\nabla \times \left( {\overline{M \times H} } \right) = \frac{1}{2}\nabla \times - 6\mu_{n} \varphi_{1} m_{1} \Omega \, = - \frac{3}{2}\mu_{n} \varphi_{1} m_{1} \nabla \left( {\nabla .V} \right) + \frac{3}{2}\mu_{n} \varphi_{1} m_{1} \nabla^{2} v = \frac{3}{2}\mu_{n} \varphi_{1} m_{1} \nabla^{2} V $$

From (8) the rotational viscosity owing to the magnetic field is $\left( {\frac{3}{2}\mu_{n} \varphi_{1} m_{1} } \right)$. From (6) and (8)

9 $$ \rho_{n} \left[ {V_{t} + \left( {V.\nabla } \right)V} \right] = - \nabla \tilde{p} + \mu_{n} \left( {1 + \frac{3}{2}\varphi_{1} m_{1} } \right)\nabla^{2} V $$

The physical properties of the ferrofluid are as follows^40,41^:

10 $$ \rho_{n} = \left( {1 - \varphi_{1} } \right)\rho_{f} + \varphi_{1} \rho_{s} ,\,\mu_{n} = \frac{{\mu_{f} }}{{\left( {1 - \varphi_{1} } \right)^{2.5} }} $$

The density of the nanofluid is determined by the density of the base fluid as well as the volume concentration. The density of the nanofluid improves as the concentration of iron (III) oxide raises. It also affects the viscosity of nanofluid.

Because the flow is assumed to be constant and axisymmetric, Eqs. (1) and (9) may be expressed in cylindrical coordinates as^4,13,40^:

11 $$ u_{r} + \frac{u}{r} + w_{z} = 0 $$

12 $$ \rho_{n} \left[ {uu_{r} + wu_{z} - \frac{{v^{2} }}{r}} \right] = - \tilde{p}_{r} + \mu_{n} \left( {1 + \frac{3}{2}\varphi_{1} m_{1} } \right)\left\{ {u_{zz} + \frac{1}{r}u_{r} - \frac{u}{{r^{2} }} + u_{rr} } \right\} $$

13 $$ \rho_{n} \left[ {uv_{r} + wv_{z} + \frac{uv}{r}} \right] = \mu_{n} \left( {1 + \frac{3}{2}\varphi_{1} m_{1} } \right)\left\{ {v_{zz} + \frac{1}{r}v_{r} + v_{rr} - \frac{v}{{r^{2} }}} \right\} $$

14 $$ \rho_{n} \left[ {uw_{r} + ww_{z} } \right] = - \tilde{p}_{z} + \mu_{n} \left( {1 + \frac{3}{2}\varphi_{1} m_{1} } \right)\left\{ {w_{zz} + \frac{1}{r}w_{r} + w_{rr} } \right\} $$

The boundary conditions are

15 $$ \left. \begin{gathered} z = 0,u = 0,v = r\omega ,w = 0, \hfill \\ z \to \infty ,u \to 0,v \to 0 \hfill \\ \end{gathered} \right\}. $$

In this case, the disk rotates with a constant angular velocity along an axis parallel to its plane. The flow takes into account the demands of no-slip boundaries as suggested by Animasaun et al.^24^. At the disc, the nanofluid layer is carried with it. This thin layer of nanoparticles is pushed outward by centrifugal force. Due to centrifugal force, fresh fluid particles are ejected as they move toward the disc in an axial direction. The nano-particles are kept safely away from the wall by combining the centrifugal force and the radial pressure gradient.

### Numerical approach

The swirling flow of a water-based Fe_3_O_4_ nanofluid has been modeled in the current problem using COMSOL Metaphysics' computational fluid dynamics (CFD) module 2D axisymmetric module. In the answer, the following physical parameters were used:

The domain has eight bounds, as seen in Fig. 1b. The highest element size used is $\left( {9 \times 10^{ - 4} } \right)$, while the $\left( {4 \times 10^{ - 5} } \right)$ smallest element size is. The chosen curvature factor is 0.3, and the maximum element growth rate is 1.15. For various volume fractions and effective magnetic parameters, surface charts of velocity magnitude, radial, tangential, and axial velocity on the nanofluid are provided. Table 1 shows the values of the variables applied in the solution. Quadratic extrapolation functions are utilized in the CFD Module to approximate the results. The issue is solved using triangular and quadrilateral components. Table 2 displays the mesh description of the components as well as their characteristics. The number of generations is 300, and the solution error is in the magnitude of $\left( {10^{ - 5} } \right)$. Table 3 shows the comparison of current research work with previously published work. It shows good agreement between present and old work. Figure 1c shows the flow chart of the FEM procedure.

16 $$ \left. \begin{gathered} u = r\omega E\left( \alpha \right),\quad v = r\omega F\left( \alpha \right),\quad w = \sqrt {v_{f} \omega } G\left( \alpha \right), \hfill \\ \tilde{p} - p_{\infty } = - \rho v\omega P\left( \alpha \right),\quad \alpha = \sqrt {\frac{\omega }{{v_{f} }}} z \hfill \\ \end{gathered} \right\}, $$

17 $$ G_{\alpha } + 2E = 0 $$

18 $$ \left( {1 + \frac{3}{2}\varphi_{1} m_{1} } \right)\left[ {\frac{1}{{\left( {1 - \varphi_{1} } \right)^{2.5} \left( {1 - \varphi_{1} + \varphi \frac{{\rho_{s} }}{{\rho_{f} }}} \right)}}} \right]E_{\alpha \alpha } - GE_{\alpha } - E^{2} + F^{2} = 0 $$

19 $$ \left( {1 + \frac{3}{2}\varphi_{1} m_{1} } \right)\left[ {\frac{1}{{\left( {1 - \varphi_{1} } \right)^{2.5} \left( {1 - \varphi_{1} + \varphi \frac{{\rho_{s} }}{{\rho_{f} }}} \right)}}} \right]F_{\alpha \alpha } - GF_{\alpha } - 2EF = 0 $$

20 $$ \left( {1 + \frac{3}{2}\varphi_{1} m_{1} } \right)\left[ {\frac{1}{{\left( {1 - \varphi_{1} } \right)^{2.5} \left( {1 - \varphi_{1} + \varphi \frac{{\rho_{s} }}{{\rho_{f} }}} \right)}}} \right]G_{\alpha \alpha } - GG_{\alpha } - P_{\alpha } = 0 $$

With boundary conditions

21 $$ E\left( 0 \right) = 0,\,F\left( 0 \right) = 1,\,G\left( 0 \right) = 0,\,E\left( \infty \right) = 0,\,F\left( \infty \right) = 0\, $$

The reduced pressure is

22 $$ P\left( \alpha \right) = P_{0} + \left( {1 + \frac{3}{2}\varphi_{1} m_{1} } \right)\left[ {\frac{1}{{\left( {1 - \varphi_{1} } \right)^{2.5} \left( {1 - \varphi_{1} + \varphi \frac{{\rho_{s} }}{{\rho_{f} }}} \right)}}} \right]G_{\alpha } - \frac{1}{2}G^{2} $$

**Table 1:** Numeric values of nano-particle properties utilized in the present study^4,16^.

Parameters	$\left( {\rho_{f} } \right)$	$\left( {\rho_{s} } \right)$	$\left( {\varphi_{1} } \right)$	$\left( {\mu_{f} } \right)$	$\left( {m_{1} } \right)$	$\omega$
Values	997.1 $\left( {{\text{kg}}\;{\text{m}}^{ - 3} } \right)$	5500 $\left( {{\text{kg}}\;{\text{m}}^{ - 3} } \right)$	0.1	0.001 $\left( {{\text{Pa}}\;{\text{s}}} \right)$	2	$0.5\pi$ $\left( {{\text{rad}}\;{\text{s}}^{ - 1} } \right)$

**Table 2:** Mesh descriptions during the finite element method over the domain of the flow^15^.

Property	Minimum element	Average element	Triangular elements	Quadrilateral elements	Edge elements	Vertex elements
Value	0.07274	0.794	4679	984	265	8

**Table 3:** A similar solution is compared to earlier theoretical models $m_{1} = \varphi_{1} = 0$.

Comparison	$F^{\prime}\left( 0 \right)$	$E^{\prime}\left( 0 \right)$
Kelson and desseaux ^42^	− 0.615922	0.510233
Bachok et al. ^43^	− 0.6159	0.5102
Turkyilmazoglu ^44^	− 0.61592201	0.51023262
Bhandari ^40^	− 0.6159241	0.5102337
Current outcomes	− 0.6159262	0.5102346

## Results and discussion

In this section, different two-dimensional plots are discussed to analyze the study outcomes. The nanofluid's behavior on a rotating disk under the effect of the magnetic field has been observed for distinct study profiles.

### Radial and tangential velocity profiles

**Figure 2:**
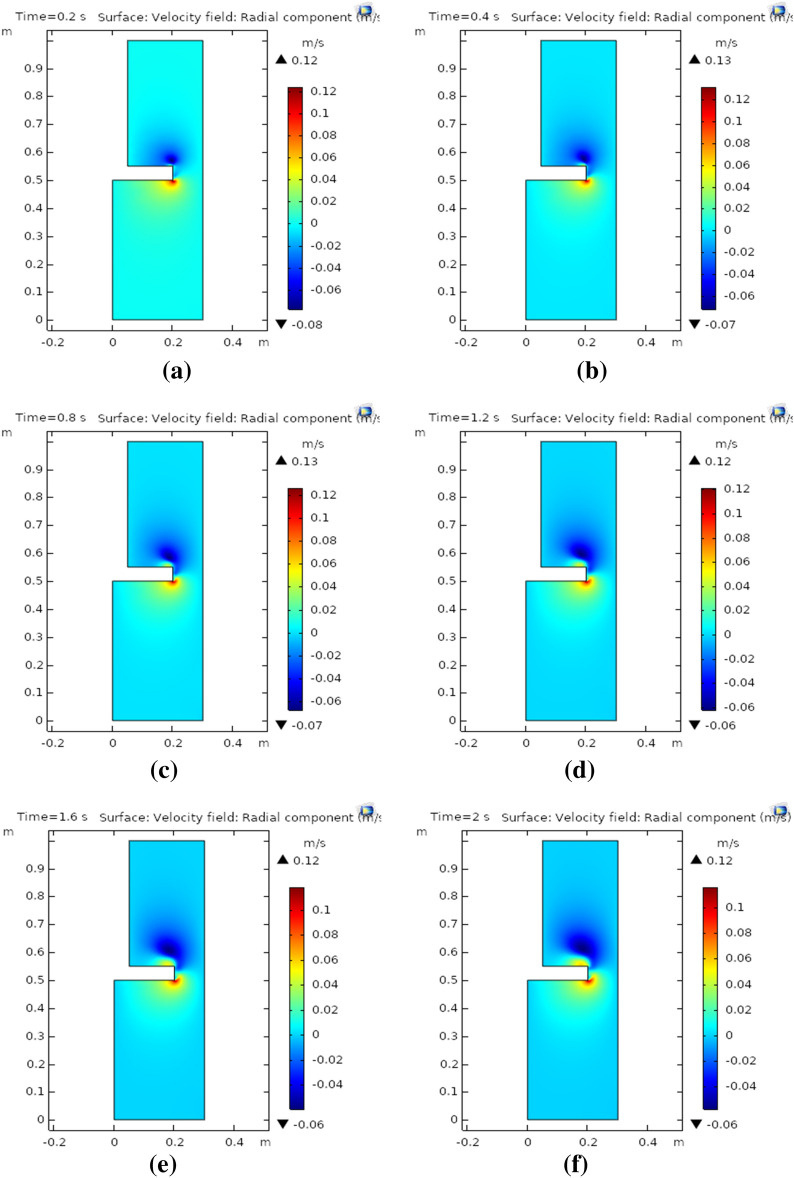
**(a)** Radial component at 0.2 s, **(b)** radial component at 0.4 s, **(c)** radial component at 0.8 s, **(d)** radial component at 1.2 s, **(e)** radial component at 1.6 s, **(f)** radial component at 2 s.

Figure 2a–f depict the velocity profile in the radial direction. The radial profile has been generated at a different set time as follows 0.2 s, 0.4 s, 0.8 s, 1.2 s, 1.6 s, and 2 s. Figure 2a shows that at time 0.2 s at the lower edge is found maximum whereas minimum radial velocity is observed at the upper edge in a two-dimensional plot. It is worth noting that as time increases to 0.4 s, the radial velocity has slightly increased (see Fig. 2b). Figure 2c, d, show the radial motion of nanofluids has been observed to decrease as time is incremented from 0.8 to 1.2 s, respectively. Swirling nanofluid motion has been observed at the upper and lower edges of the rotating disk as time is enhanced from 0.8 to 1.2 s. This effect also shows that in the near region of rotation upsurge in radial velocity profile was observed as time moves forwards. Figure 2e, f demonstrate the downfall in radial motion of rotating nanofluids, the overall velocity field has decreased up to 0.20%. The radial velocity profile has decreased with the maximum observed velocity in the radial direction as 0.1 m/s. It can be observed that as the passes radial motion. It is also concluded that pressure generated at the round corner of the disk assists the radial flow of nanofluid at the lower surface whereas resistance has been observed at the upper surface of the disk.

**Figure 3:**
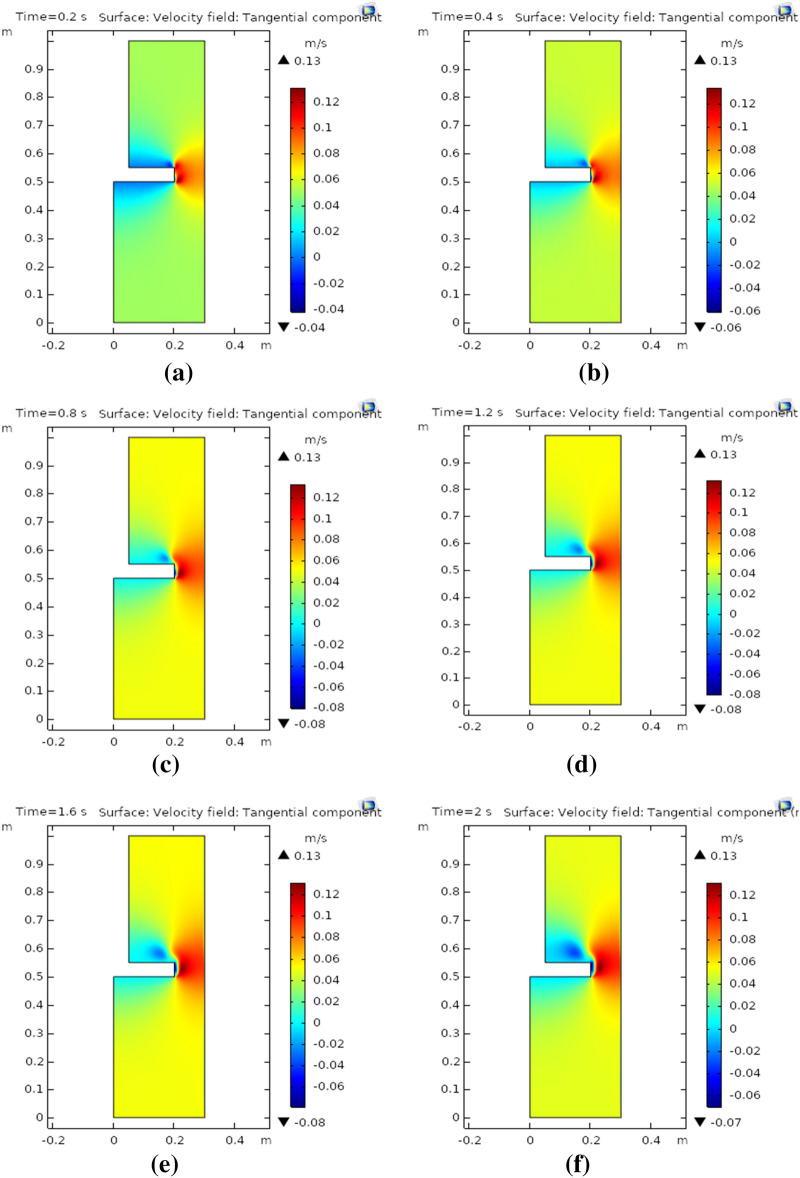
**(a)** Tangential component at 0.2 s, **(b)** tangential component at 0.4 s, **(c)** tangential component at 0.8 s, **(d)** tangential component at 1.2 s, **(e)** tangential component at 1.6 s, **(f)** tangential component at 2 s.

Tangential velocity plots for the rotational flow of nanofluid have been generated at set time scales. Figure 3a–f demonstrate the behavior of tangential velocity for 0.2 s, 0.4 s, 0.8 s, 1.2 s, 1.6 s, and 2 s, respectively. Although, maximum tangential velocity was found for times 0.2 s and 0.4 s, whereas, minimum velocity in the tangential direction was observed at the upper and lower surface of the rotating disk. Swirling motion of fluid was also attained, moreover, the rotating movement of fluid near a curved surface was slightly higher at 0.4 s as compared to 0.2 s (see Fig. 3a, b), Fig. 3c, d describe the tangential velocity profile at times 0.8 s and 1.2 s, respectively. The tangential velocity at both times takes a moderate upsurge throughout the two-dimensional plot. The maximum tangential velocity observed attained is 0.13 m/s at 0.8 s and 1.2 s. Now, as time increases the Swirling flow around the curved surface of the rotating disk is augmented. The tangential velocity profile is observed at times 1.6 s and 2 s in Fig. 3e, f, respectively. The maximum tangential velocity observed is 0.13 m/s for both times under consideration. It is worth noting that as time passes by the Swirling flow around the curved surface of the rotating disk starts expanding and at the upper surface of the disk there is no motion of free-moving nanofluid. In other words in the surrounding rotating disk, a moderate tangential velocity of 0.06 m/s is observed in the whole region.

### Pressure profile and streamlines

**Figure 4:**
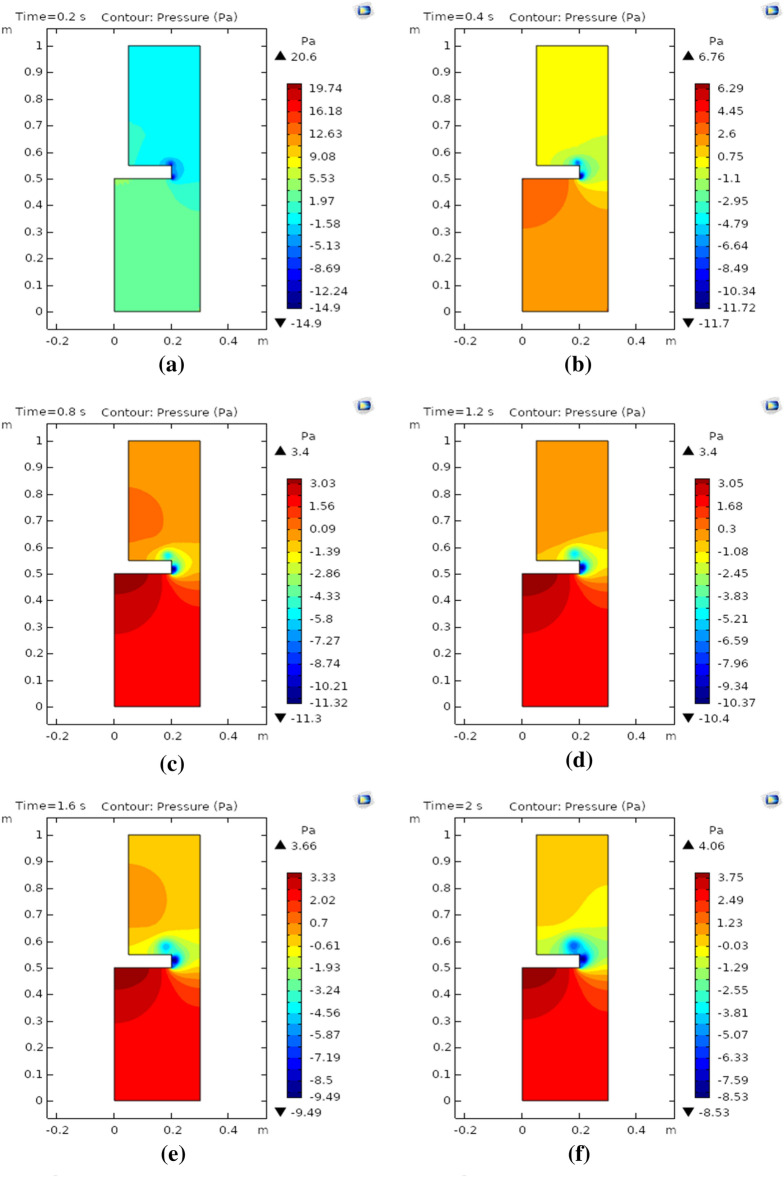
**(a)** Pressure contours at 0.2 s, (**b)** pressure contours at 0.4 s, (**c)** pressure contours at 0.8 s, (**d)** pressure contours at 1.2 s, € pressure contours at 1.6 s, (**f)** pressure contours at 2.0 s.

Figure 4a–f depict the exerted pressure on the surrounding boundaries of the rotating disk under the impression of the magnetic field at times 0.2 s, 0.4 s, 0.8 s, 1.2 s, 1.6 s, and 2 s, respectively. Figure 4a shows that when the fluid attains some motion due to the rotation of the disk the maximum exerted pressure is observed at adjacent boundaries. The obtained maximal pressure is 20.6 Pa and near the curved surface of the circular disk, there was found minimal free movement of the fluid. Figure 4b describes that with a 0.2 s increment in time step size, the pressure in rotating flow sharply drops from 20.6 to 6.76 Pa. Figure 4c, d illustrate the pressure profile of rotating nanofluid at 0.8 s and 1.2 s. It is worth noting that the pressure profile drops further, this phenomenon occurs due to the smooth rotating motion of nanofluid which not only provides a set path to nanofluid but also decreases the pressure profile abruptly. Figure 4e depicts the pressure exerted due to the rotating motion of the nanofluid at time 1.6 s. The pressure profile drops further from 6.76 to 3.66 Pa. This occurs due to Lorentz's force-generated resistance in the smooth motion of rotating nanofluids which eventually decreases the pressure profile. Figure 4f demonstrates the pressure profile at the endpoint in the time range. The pressure exerted increases slightly from 3.66 to 4.06 Pa. Moreover, the pressure profile associated with rotating nanofluid shows that at curved surfaces rotating disk minimal pressure is observed while at the lower surface of the rotating disk, the pressure exerted is maximum.

**Figure 5:**
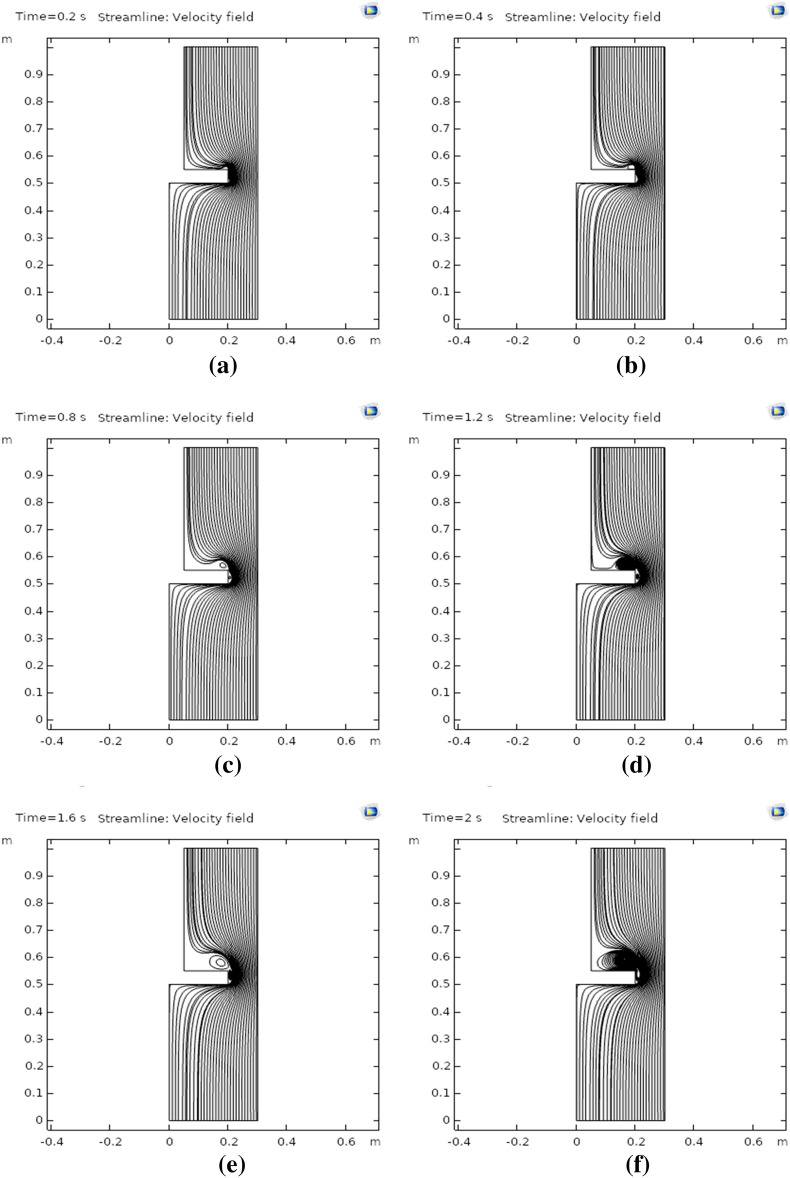
**(a)** Streamlines at 0.2 s, **(b)** streamlines at 0.4 s, **(c)** streamlines at 0.8 s, **(d)** streamlines at 1.2 s, **(e)** streamlines at 1.6 s, **(f)** streamlines at 2.0 s.

Figure 5a–f are generated to illustrate the streamlines of rotating nanofluid flow at times 0.2 s, 0.4 s, 0.8 s, 1.2 s, 1.6 s, and 2 s, respectively. The streamlines near the curved surface of the rotating disk were observed as dense. Figure 5a, b, describe the very smooth pattern of streamlines at times 0.2 s and 0.4 s, respectively. Figure 5c, d point out that around the curved surface of the disk and at the upper plain surface swirling patterns are observed for streamlines. Figure 5e demonstrates that the swirling pattern of streamlines has increased with an increment in time step size. Figure 5f showed that as we increased the time the swirling pattern of streamlines grows denser and denser. Moreover, the streamlines were set to magnitude controlled and uniformly distributed in the whole domain under observation. The total number of streamlines utilizes to study the flow patterns of streamlines was adjusted to forty. The uniform distribution scale would disperse the total number of streamlines into the whole domain equally spaced.

### Three-dimensional surface velocity magnitude

**Figure 6:**
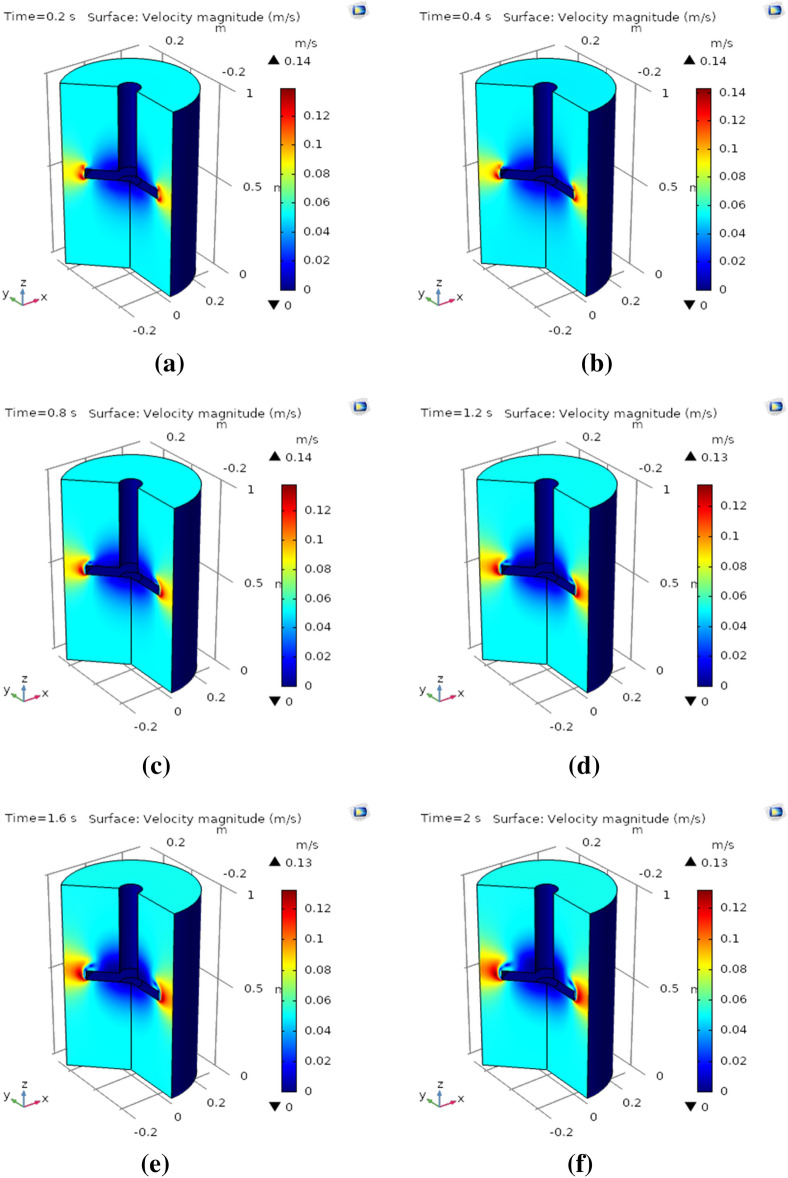
** (a)** Velocity magnitude at 0.2 s in 3D, **(b)** velocity magnitude at 0.4 s in 3D, **(c)** velocity magnitude at 0.8 s in 3D, **(d)** velocity magnitude at 1.2 s in 3D, **(e)** velocity magnitude at 1.6 s in3D, **(f)** velocity magnitude at 2.0 s in 3D.

The three-dimensional plots are generated to demonstrate the behavior of the surface velocity magnitude profile. Figure 6a–f describe the behavior of nanofluid over a rotating disk in the three-dimensional system at 0.2 s, 0.4 s, 0.8 s, 1.2 s, 1.6 s, and 2.0 s, respectively. Figure 6a shows that at the upper and lower surface of the rotating disk, the minimal motion of nanofluid is attained, and around the curved surface of the disk maximum velocity is achieved. Figure 6b, c explain the surface velocity magnitude; the maximum achieved velocity is 0.14 m/s at 0.4 s and 0.8 s, respectively. It is worth noting that at a curved surface, the maximum magnitude of the velocity profile is achieved. Swirling motion at the upper and lower surface also increases as the time varies. Figure 6d discloses that the magnitude of the velocity profile slightly falls by 0.01%. Figure 6e, f illustrate surface magnitude at 1.6 s and 2 s. These graphs show that at the upper and lower surface, the swirling rotating nanofluid enhances as time varies and along with a curved surface maximum velocity magnitude profile is achieved. The maximum obtain surface velocity magnitude is 0.12 m/s at both times 1.6 s and 2.0 s, respectively. Moreover, the overall surface velocity magnitude in the whole domain is observed to be around at all times around 0.06 m/s.

### Discrete upper surface velocity magnitude

**Figure 7:**
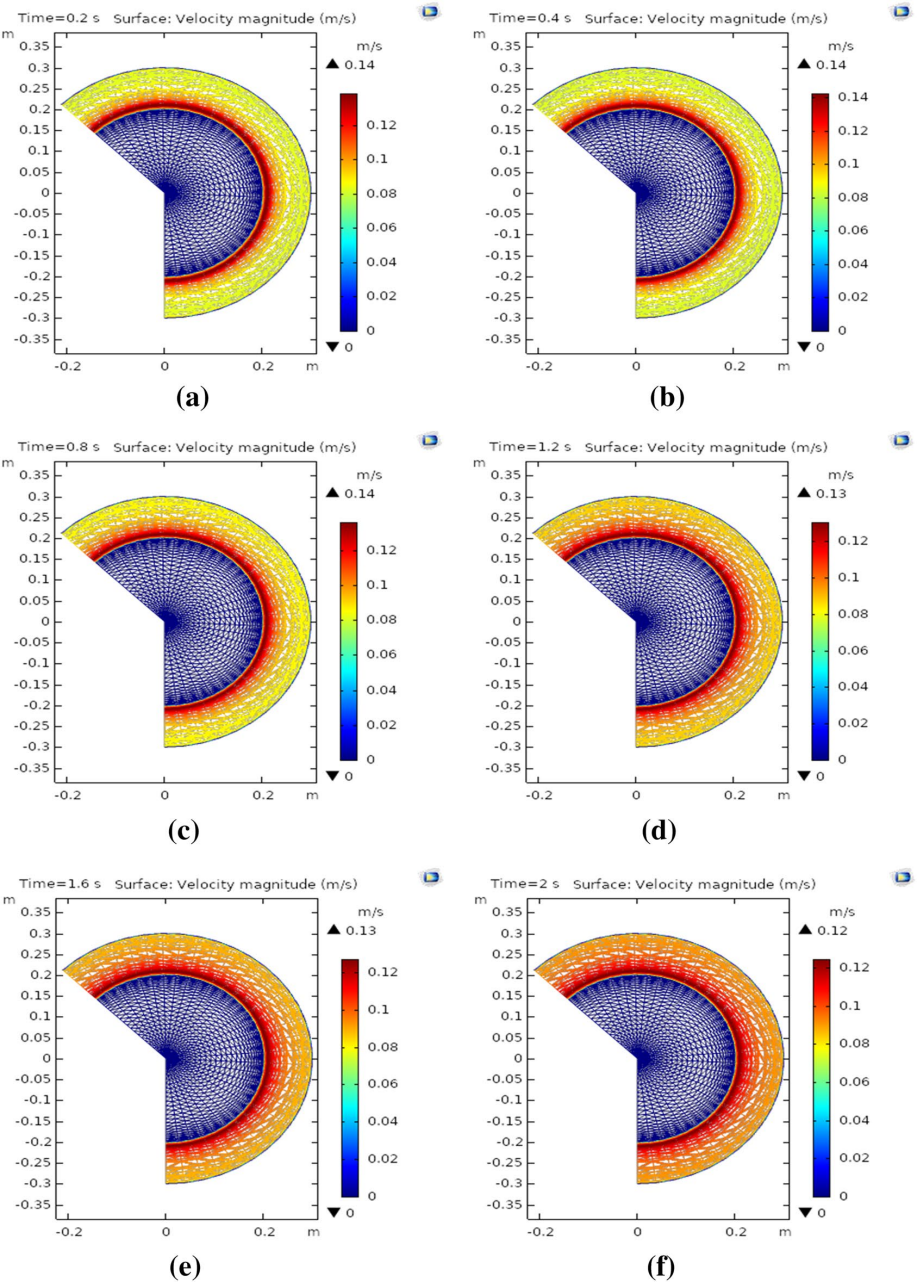
**(a)** Velocity magnitude of the disk at 0.2 s, **(b)** velocity magnitude of the disk at 0.4 s, **(c)** velocity magnitude of the disk at 0.8 s, **(d)** velocity magnitude of the disk at 1.2 s, **(e)** velocity magnitude of the disk at 1.6 s, **(f)** velocity magnitude of the disk at 2 s.

The change in surface velocity magnitude at the upper surface of the rotating disk is presented in Fig. 7a–f at respective times. These upper surface plots also disclose the detailed change in velocity magnitude with a variation of the time step. Figure 7a, b showed that the maximum surface magnitude at the upper surface of the disk is 0.14 m/s at times 0.2 s and 0.4 s, respectively. It is also worth noting here that the change in surface magnitude is visible. The blue region indicates minimal velocity, the green region denotes moderate surface magnitude and the red zone demonstrates maximal surface velocity magnitude for both times 0.2 s and 0.4 s. Figure 7c, d depict that surface magnitude at the upper surface, the surface magnitude has decreased by 0.01% at time 1.2 s as compared to 0.8 s. The patterns observed for surface velocity magnitude are distinct and clear to observe and analyze the change in velocity magnitude with varying times. Figure 7e, f describe the surface magnitude at the upper surface of the rotating disk at times 1.6 s and 2 s, respectively. It is worth mentioning that the maximum surface velocity magnitude achieved is 0.12 m/s at times 1.6 s and 2 s. Moreover, the red region starts increasing with varying times describing the increment in surface velocity magnitude.

**Figure 8:**
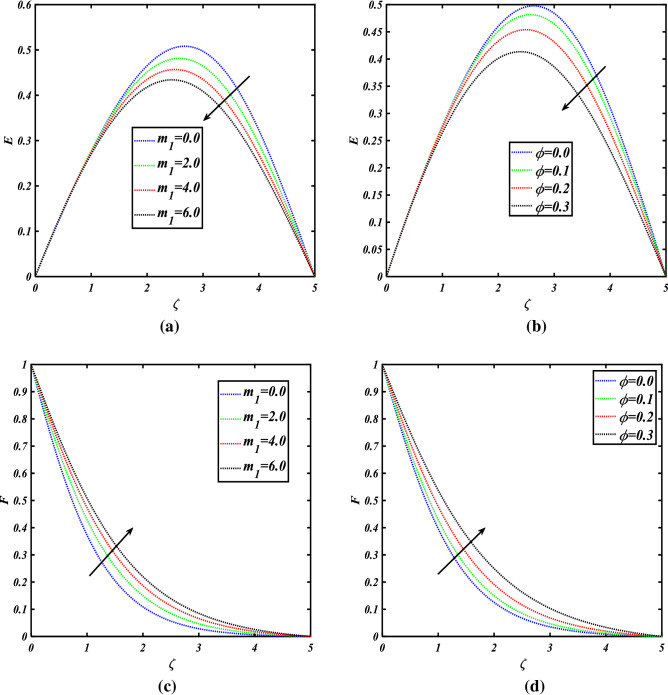

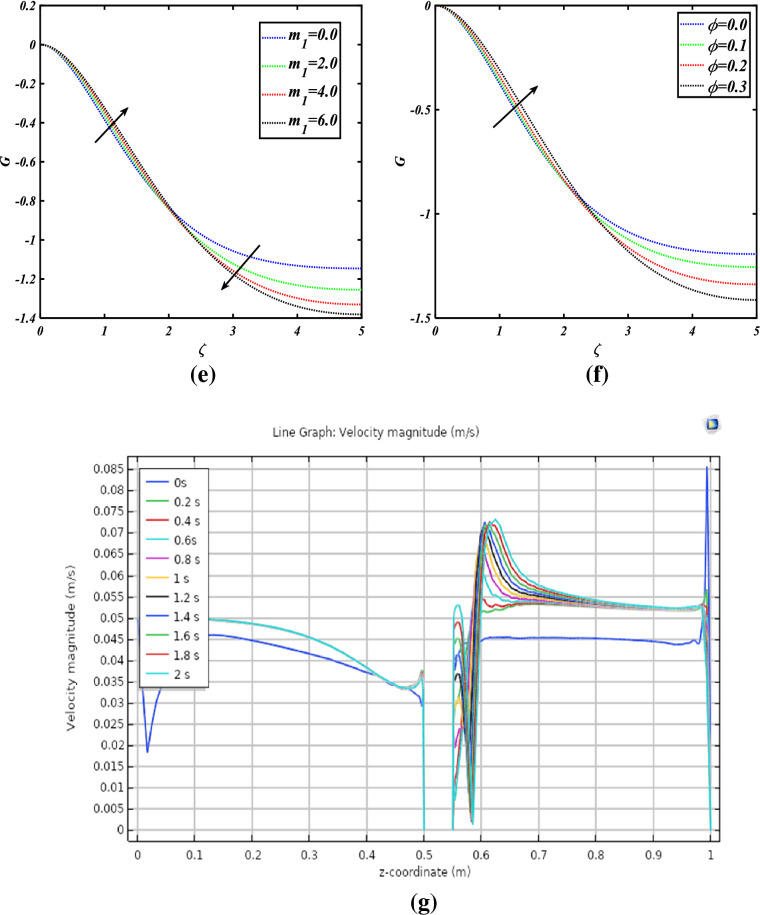
**(a)** Radial velocity for the values of $m_{1}$, **(b)** radial velocity for the values of $\phi$, **(c)** tangential velocity for values of $m_{1}$, **(d)** tangential velocity for values of $\phi$, **(e)** axial velocity for the values of $m_{1}$, **(f)** axial velocity for the values of $\phi$, **(g)** the effects of a line graph.

Figure 8a, b show the importance of the radial velocity field for the numerous values $m_{1} \& \phi$. The radial velocity field is decreased for the increasing values of both parameters $m_{1} \& \phi$. Figure 8c, d show the importance of the tangential velocity profile for the numerous values $m_{1} \& \phi$. The tangential velocity profile is boosted for the increasing values of both parameters $m_{1} \& \phi$. Figure 8e, f show the importance of the axial velocity profile for the numerous values $m_{1} \& \phi$. The axial velocity profile is enhanced for the increasing values of both parameters $m_{1} \& \phi$.

### Line graph

The graph is plotted to illustrate the overall behavior of the velocity profile at every step size of time (Fig. 8g). The velocity magnitude in normal to the direction of rotation is obtained and the abrupt downfall right in the center is due to the rotating disk. It is observed that after passing the rotating disk expansion in the momentum boundary layer has been observed as the time varies. The taken time step size is 0.2 s, if a smaller step size is selected then more exclusive and miscellaneous patterns would have been obtained.

### Validation of results

**Figure 9:**
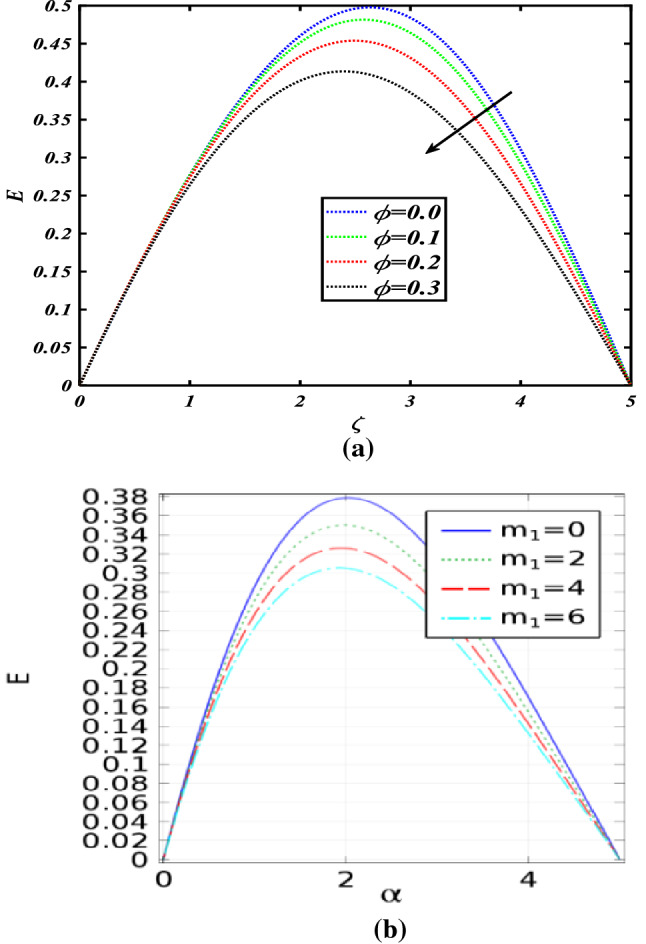
** (a)** Radial velocity under the influence of nano-particle concentration profile, (**b**) results obtained by^40^ for radial velocity under the varying concentration of nano-particles.

Present outcomes have been validated (Fig. 9a) with Bhandari et al.^40^ under the varying influence of nano-particle concentration (Fig. 9b). The results obtained in this study are in complete agreement with already published previous results. Furthermore, the results achieved in this study are more accurate, and correct and show the effectiveness of the proposed methodology utilized in this study.

### Final remarks

In this article, the role of nano-particle $\left( {{\text{Fe}}_{3} {\text{O}}_{4} } \right)$ in heat transfer enhancement over a rotating disk has been investigated. Water has been used as the base fluid in the present study. The computational fluid dynamics approach is utilized in the present analysis to illustrate the axisymmetric flow of nanofluid. In addition, for our convenience, we have taken into account the two-dimensional configuration of axisymmetric flow to discuss varying radial and tangential velocities profile over different time steps. Additionally, the effect of nanoparticle concentration and magnetization force has been discussed on radial and tangential velocities. Furthermore, the present outcomes of the study have been validated with already published previous results and were found in complete agreement. According to the results, the inclusion of $\left( {{\text{Fe}}_{3} {\text{O}}_{4} } \right)$ nano-material reduced overheating and favored the maximum velocity. The following were some of the primary findings:

According to these findings, enhancing the volume concentration and rotating viscosity reduces the velocity field.

These are the findings of a comparison between the computational fluid dynamics (CFD) module and dimensionless research.

Both strategies benefit from each other's results. The dimensionless analysis is critical for the physical analysis of the findings. However, the computational fluid dynamics (CFD) module is essential for real-time particle tracing.

These findings indicate that both types of research are important for the advancement of swirling flow analysis.

These findings might help to enhance the sealing of hard disk devices.

## Data Availability

The datasets used and analyzed during the current study are available from the corresponding author upon reasonable request.
